# Cerebellar Development and Circuit Maturation: A Common Framework for Spinocerebellar Ataxias

**DOI:** 10.3389/fnins.2020.00293

**Published:** 2020-04-02

**Authors:** Francesca Binda, Carla Pernaci, Smita Saxena

**Affiliations:** ^1^Department of Neurology, Center for Experimental Neurology, University Hospital of Bern, Bern, Switzerland; ^2^Department for BioMedical Research (DBMR), University of Bern, Bern, Switzerland; ^3^Graduate School for Cellular and Biomedical Sciences, University of Bern, Switzerland

**Keywords:** cerebellum, spinocerebellar ataxia, Purkinje cell degeneration, cerebellar circuits, circuit maturation

## Abstract

Spinocerebellar ataxias (SCAs) affect the cerebellum and its afferent and efferent systems that degenerate during disease progression. In the cerebellum, Purkinje cells (PCs) are the most vulnerable and their prominent loss in the late phase of the pathology is the main characteristic of these neurodegenerative diseases. Despite the constant advancement in the discovery of affected molecules and cellular pathways, a comprehensive description of the events leading to the development of motor impairment and degeneration is still lacking. However, in the last years the possible causal role for altered cerebellar development and neuronal circuit wiring in SCAs has been emerging. Not only wiring and synaptic transmission deficits are a common trait of SCAs, but also preventing the expression of the mutant protein during cerebellar development seems to exert a protective role. By discussing this tight relationship between cerebellar development and SCAs, in this review, we aim to highlight the importance of cerebellar circuitry for the investigation of SCAs.

## Introduction

Spinocerebellar ataxias (SCAs) are a large family of movement disorders characterized by the progressive loss of motor coordination, muscle tone and control, with a broad spectrum of severity ranging from mild gait and posture problems to death. Moreover, several comorbidities including pyramidal features, peripheral neuropathy, tremor, dystonia, Parkinsonian features, myoclonus, epilepsy, and dementia occur frequently in SCAs. The 47 as of now identified SCAs are dominantly inherited diseases, and a growing number of genes are implicated, revealing two major pathological alterations; (1) Microsatellite repeat expansions coding for CAG generating polyglutamine (PolyQ) repeats: SCA1, SCA2, SCA3, SCA6, SCA7, SCA17 and Dentatorubral-pallidoluysian atrophy DRPLA, and non-coding repeats: SCA8, SCA10, SCA12, SCA31, SCA36, SCA37). (2) Single-gene point mutations as observed in SCA5, SCA11, SCA13-16, SCA18, SCA19/22, SCA20-23, SCA26, SCA27-29, SCA34-35, SCA38, SCA40-46 (Ashizawa et al., 2018; Pilotto and Saxena, 2018).

Genes causing SCAs code for a large variety of proteins including ion channels (Riess et al., 1997; Zhuchenko et al., 1997; Waters et al., 2006; Lee et al., 2012; Coutelier et al., 2015; Fogel et al., 2015; Morino et al., 2015), transcription factors and repressors (Orr et al., 1993; Koide et al., 1999; Lin et al., 2018), scaffolding proteins (Ikeda et al., 2006; Tsoi et al., 2014), or signaling kinases (Chen et al., 2003; Houlden et al., 2007), phosphatases (Holmes et al., 1999), and receptors (Storey et al., 2001; Hara et al., 2004; Hara et al., 2008; Huang et al., 2012; Watson et al., 2017). Despite this heterogeneity, SCA patients share a slow dramatic degeneration of the cerebellum and its afferent and efferent systems, suggesting a common point of interception, wherein SCA causing mutations converge and promotes the onset of these neurodegenerative diseases. Interestingly, wiring defects of the cerebellar circuitry are a common finding in SCAs and they appear at the early asymptomatic phase (Ebner et al., 2013; Hansen et al., 2013; Dulneva et al., 2015; Jayabal et al., 2017). In preclinical rodent models of SCA1, inhibiting the expression of the mutant ATXN1 protein during cerebellar postnatal development exerted a protective effect on the ataxic phenotype (Serra et al., 2006) and ameliorated synaptic transmission in the cerebellar cortex (Zu et al., 2004; Barnes et al., 2011). Therefore, cerebellar development seems to be highly vulnerable to the expression of mutant SCA-causing molecules, and the resulting compromised cerebellar compartmentation, patterning and circuit wiring could have a direct impact on the severity of the disease. In this review, we focus on the possible role of SCA genes in cerebellar development in an attempt to define a new framework for SCAs.

## The Cerebellum

The cerebellum is traditionally known for its role in motor control and more recently its involvement in higher cognitive functions has been recognized (Koziol et al., 2014; Adamaszek et al., 2017).

The cerebellum receives two major excitatory inputs: mossy fibers (MFs) and climbing fibers (CFs). MFs originate from several pre-cerebellar nuclei in the brainstem and spinal cord, and they carry vestibular, motor, sensory and proprioceptive information relayed by the granule cells (GCs). GC axons ascend into the molecular layer (ML), where they bifurcate into parallel fibers (PFs) that run longitudinally to the cerebellar lobule, contacting several Purkinje cells (PCs) on their way. Each PC receives up to 10^5^–10^6^ excitatory PF synaptic contacts, however, one PF makes only one to two synapses onto a single PC (Napper and Harvey, 1988). PF activity induces simple spike discharge in PCs; moreover, PCs spontaneously fire action potentials, and this pacemaker activity has been recorded in cerebellar slices during the pharmacological blockade of excitatory inputs (Hausser and Clark, 1997) and in cultured PCs (Nam and Hockberger, 1997). Notably, CFs only originate from the inferior olive nuclei (IO) in the brainstem and establish monoinnervation on mature PCs. CF activation signature in PCs is a high frequency burst of spikes known as complex spike (Eccles et al., 1966). CFs provide teaching signals to the cerebellum that drive cerebellar-mediated motor learning. The simultaneous activation of CF and PFs leads to long-term depression at the PF to PC synapses (Ito et al., 1982), while the sole activation of GCs promotes long-term potentiation at these synapses (Lev-Ram et al., 2002; Coesmans et al., 2004). Long-term plasticity at these excitatory synapses provides a major molecular platform for supporting cerebellar-mediated motor learning (De Zeeuw et al., 1998; Boyden et al., 2006; Hansel et al., 2006; Schonewille et al., 2010; Ly et al., 2013). Traditionally, CFs are known to carry sensory/motor errors but more recently, they have also been shown to mediate reward and predictive signals (Ohmae and Medina, 2015; Heffley et al., 2018; Kostadinov et al., 2019; Larry et al., 2019).

**FIGURE 1:**
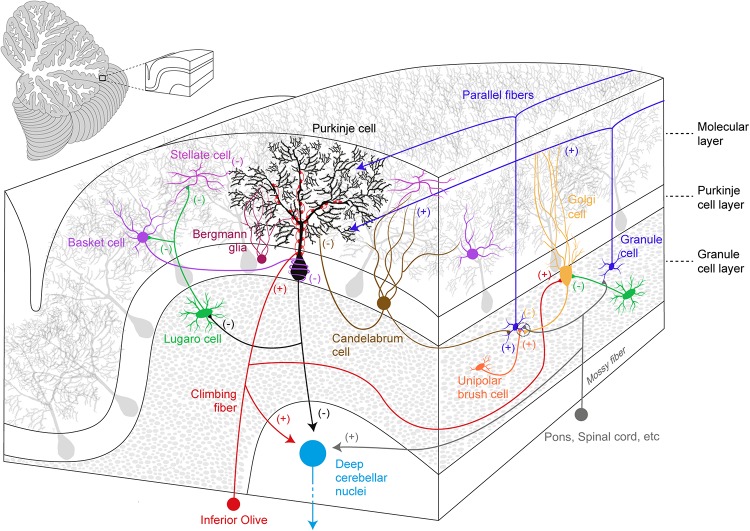
Simplified overview of the cerebellar cortical organization and its circuitry. Excitatory (+) and inhibitory (–) components are represented within the cerebellar cortex and deep cerebellar nuclei.

Anatomically, three parts are recognized in the cerebellum: the cerebellar cortex, the white matter and the deep cerebellar nuclei (DCN). The cerebellar cortex is the most superficial structure and organized into three layers (from the most superficial): the ML, the Purkinje cell layer (PCL), and the granule cell layer (GCL) (Figure 1).

The ML accommodates the complex and ramified dendritic tree of PCs together with two types of GABAergic interneurons (molecular layer interneurons, MLIs): Basket cells (BCs) and Stellate cells (SCs). BCs synapse onto PC soma and its axon initial segment in a characteristic structure known as *pinceau* (Ango et al., 2004; Blot and Barbour, 2014), while SCs innervate PC dendrites. MLIs receive excitatory inputs from PFs and they provide feed-forward inhibition, which modulates PC spike output (Mittmann et al., 2005; Brown et al., 2019), calcium influx and long-term plasticity (Binda et al., 2016). Moreover, CF activation also recruits MLIs via a spillover mechanism (Szapiro and Barbour, 2007). PC somas define the PCL: PCs are the sole output of the cerebellar cortex and they send GABAergic projections to the DCN and vestibular nuclei. The PCL also hosts the small vertically oriented pear-shaped soma of Candelabrum cells (CaCs). CaCs have one or two long dendrites that enter the ML, and several short dendrites localized below the GCL. The axon of these neurons runs horizontally and is characterized by multiple vertical branches ascending to the ML (Laine and Axelrad, 1994). In the *Macaca* monkey, the immunohistochemical analysis of the cerebellum provided evidence for the release of glycine and GABA neurotransmitters by these neurons (Crook et al., 2006), however, CaCs targets and inputs still remain elusive.

Together with GCs, the GCL also contains glutamatergic interneurons, the Unipolar Brush cells (UBCs) that establish excitatory inputs onto GCs and on other UBCs (Nunzi et al., 2001). UBCs mainly receive excitatory inputs from a single MF carrying vestibular information and they are particularly enriched in the posterior cerebellum and flocculus (Rossi et al., 1995). UBCs also receive inhibitory synaptic inputs from Golgi Cells (GoCs) (Dugue et al., 2005). Glycinergic/GABAergic interneurons also populate the GCL and these include GoCs and Lugaro Cells (LCs). GoCs provide the sole inhibitory input to GCs, via feed-forward and a feedback inhibitory loops (Kanichay and Silver, 2008). LCs have spindle-shaped cell bodies (Laine and Axelrad, 1996) and they innervate the MLIs (Laine and Axelrad, 1998), GoCs (Dieudonne and Dumoulin, 2000; Dumoulin et al., 2001), and PCs (Dean et al., 2003). A prominent feature of these interneurons is their activation by serotonin, which induces firing in an otherwise silent cell (Dieudonne and Dumoulin, 2000; Dumoulin et al., 2001).

## Purkinje Cells Development, Connectivity and Functional Deficits in SCAs

PCs originate in the ventricular zone around embryonic day E10.5-E13.5 in the mouse; by E14, they form a multicellular layer called the PC plate, a temporary structure which is replaced by a monolayer organization at early postnatal stage. PCs are characterized by an extensive dendritic tree, which develops during the first three postnatal weeks in the mouse (Sotelo and Dusart, 2009). The expression or the absence of specific molecular markers in PCs (Ex. ZebrinII (ZII)/Aldolase C) defines the compartmentation of the cerebellar cortex, organized in an alternate series of positive/negative parasagittal PC stripes (Brochu et al., 1990). The timing of PC birth determines their molecular fate, with early born (E10-E11.5) PCs becoming ZII+ cells and late-born (E11.5-E13.5) PCs destined to have a ZII− phenotype (Miale and Sidman, 1961). The spatial arrangement of CFs and MFs terminals follow PC patterning in the cerebellar cortex. Notably, immunolabelling of IO subnuclei in the rat revealed the preferential targeting of strongly ZII+ parasagittal bands by CFs originating from the subnucleus a of the caudal medial accessory olive (c-MAO), the rostral part of the MAO (r-MAO) and the dorsal and ventral lamellas of the principal olive (d-PO and v-PO, respectively). In contrast, ZII− bands of PCs are targeted by the subnucleus b of the c-MAO (Sugihara and Shinoda, 2004). Also, spinocerebellar and cuneocerebellar MFs terminate in the anterior cerebellum in complementary parasagittal bands with a defined distribution relative to the ZII± bands in this region (Ji and Hawkes, 1994; Valera et al., 2016). PC patterning plays a role in the definition of the final topographic map of cerebellar excitatory afferents and its modifications are mirrored by the altered spatial arrangement of CF and MF terminals (Blatt and Eisenman, 1988; Sillitoe et al., 2010; Reeber et al., 2013).

Activity-dependent mechanisms influence cerebellar compartmentation, the fine delineation of afferents topographic map and circuitry wiring. Of note, the targeted silencing of PC-specific neurotransmission affected the organization of ZII stripes, and altered spinocerebellar MF patterning (White et al., 2014). Furthermore, monoinnervation of PCs via CF relies on activity-dependent calcium influx as revealed by the impairment in the biased strengthening of a single CF in Ca_v_2.1 (P/Q type) PC-targeted knockout (KO) mice (Hashimoto et al., 2011; Kawamura et al., 2013).

Purkinje cells are also involved in the final maturation of the cerebellum: during the early postnatal period, PC-mediated Sonic Hedgehog (SHH) signaling drives the proliferation of GC progenitors within the External Granule cell Layer (EGL) (Wechsler-Reya and Scott, 1999) and the level of SHH signaling is an important determinant for cerebellar foliation (Corrales et al., 2006).

Several SCA-linked mutations directly or indirectly affect PC activity, calcium dynamics and dendrite development. They are therefore potentially harmful to cerebellar compartmentation, wiring and afferent organization.

PC firing defects have been described in several SCA models. The expression of the mutant ATXN1 in SCA1 PCs or ATXN2 in SCA2 PCs reduced their firing frequency (Hansen et al., 2011; Dell’Orco et al., 2015) and this decrease was also associated with an increase in PC firing variability in the SCA2 mouse model (Kasumu et al., 2012). Furthermore, *in vivo* analysis of complex spike activity in SCA2 mice revealed a reduction in the complex spike frequency in response to pharmacological IO stimulation by systemic harmaline injection (Egorova et al., 2018).

Deletion or mutation in SPTBN2 gene (β-III spectrin) (Ikeda et al., 2006) causes SCA5, and β-III knockout in mice resulted in transient and resurgent sodium current reduction in PCs, leading to a decrease in PC spontaneous firing. Furthermore, PC single spike frequency was altered while no difference in complex spikes was detected (Perkins et al., 2010). Moreover, PCs presented reduced dendritic surface area, impaired mono-planar dendritic arborization and reduced spine density (Gao et al., 2011). An impaired dendritic development and spine density reduction is also a feature of SCA14 as demonstrated in cultured PCs expressing PKCγ harboring the SCA14-causing S119P mutation (Chen et al., 2003; Seki et al., 2009).

SCA6 is caused by a PolyQ tract expansion in the alternatively spliced exon 47 of the *CACNA1A* gene encoding for the α1A subunit of Ca_v_2.1 voltage gated calcium channels (Zhuchenko et al., 1997). The PolyQ expansion alters Ca_v_2.1 physiology via decreased channel expression in PCs, concomitantly reducing P/Q type calcium channel-mediated currents in SCA6 knock-in mice model. These modifications, however, were independent from the length of the PolyQ expansion (Watase et al., 2008). The presence of an internal ribosome entry site (IRES) within the Ca_v_2.1 mRNA permits the expression of a C-terminus fragment (CT) containing the PolyQ tract. While the CT carrying the normal PolyQ tract serves as a transcription factor in PCs and promotes neurite outgrowth, the expression of the CT with the extended pathogenic PolyQ causes gait abnormalities and significant ML thinning (Du et al., 2013). Furthermore, *in vivo* recordings support a higher incidence of irregular PC firing in mice specifically expressing the pathogenic CT in PCs (Mark et al., 2015).

Three KCNC3 single mutations (R420H, R423H, and F448L) have been identified in families affected by SCA13 (Waters et al., 2006; Figueroa et al., 2011). KCNC3 encodes for K_v_3.3 potassium channel, expressed in PCs where it participates in CF-mediated complex spike formation (Zagha et al., 2008). Additionally, based on computer modeling, K_v_3.3-mediated conductance is proposed to interact with resurgent sodium currents in order to drive PC spontaneous firing (Akemann and Knopfel, 2006). R420H substitution in the S4 helix of K_v_3.3 leads to the loss of the channel-mediated current via a dominant negative effect (Waters et al., 2006), while R423H and F448L mutations affect the gating properties of the channel. In the K_v_3.3 KO SCA13 mouse model, PCs exhibited reduced spontaneous firing rate (Akemann and Knopfel, 2006) and lentiviral-mediated expression of K_v_3.3 R424H (murine homolog of human K_v_3.3 R423H) in cultured PCs led to impaired dendritic development (Irie et al., 2014). The missense mutation F145S in the Fibroblast Growth Factor 14 (FGF14) causes SCA27 in humans through a dominant negative effect (van Swieten et al., 2003; Brusse et al., 2006), and FGF14 ablation induces ataxia in mice (Wang et al., 2002). FGF14 modulates resurgent sodium currents in PCs (Yan et al., 2014) and FGF14 KO mice present a higher number of PCs lacking spontaneous firing when compared to WT (Shakkottai et al., 2009).

## Alterations in Circuit Wiring in the Cerebellar Cortex of SCAs

The cerebellum undergoes a profound change in terms of maturation and synaptic refinement during the first postnatal weeks, a process mediated by the activity of several molecules. During this period, CFs and PFs establish their final innervation territory onto PCs. Electrophysiological studies in mice revealed that at postnatal day 3 (P3), PC soma display multiple/polyinnervation by CFs. At this stage of development, these multiple CF synapses display similar synaptic strength, but thereafter they undergo functional differentiation. This diversification plateaus at P7 with PCs receiving both a strong CF input and several weaker ones. The weaker CFs are characterized by a lower multivesicular release probability. P/Q type voltage-gated calcium channel activity is required for CF diversification; Ca_v_2.1 deletion in PCs prevents the selective biased strengthening of a single CF, and multiple CF innervation onto PCs persists throughout the cerebellar development (Hashimoto et al., 2011; Kawamura et al., 2013). All CFs at this stage synapse onto PC soma and only by P9-10, the strongest CF, starts to translocate from the soma to PC dendrites (Hashimoto et al., 2009). Following the functional differentiation and as cerebellar development proceeds, the strong/winner CF is further strengthened via an anterograde signaling involving the C1ql1–Bai3 (brain-specific angiogenesis Inhibitor 3) pathway (Kakegawa et al., 2015). Moreover, retrograde signaling mediated by the secreted semaphorin 3A (Sema3A)–Plexin A4 and Progranulin–Sortilin 1 pathways participate in the strengthening and/or the maintenance of CFs inputs (Uesaka et al., 2014; Uesaka et al., 2018). By the end of the second postnatal week, the majority of PCs (60%) show monoinnervation by a strong CF while the remaining PCs display polyinnervation by a strong CF and one or more weaker CFs (Hashimoto and Kano, 2003). At this time point, while the winner CF proceeds further onto the PC dendritic tree, somatic synapses of weak CFs are progressively eliminated (Hashimoto et al., 2009).

**FIGURE 2:**
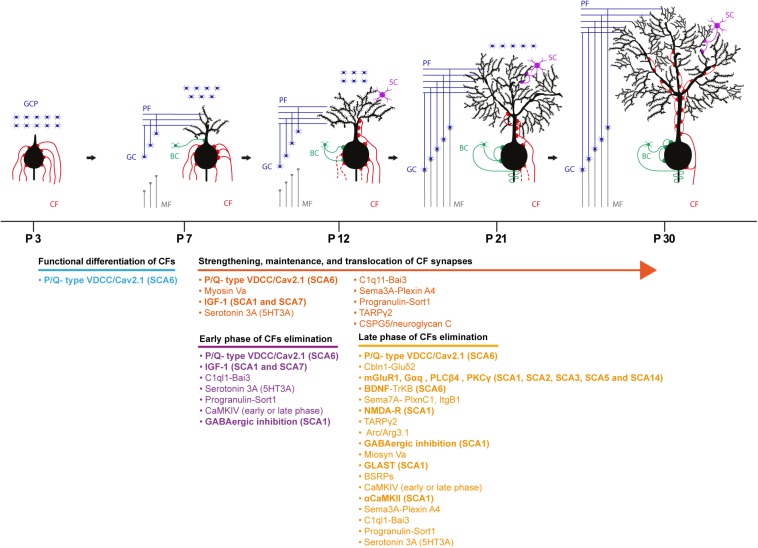
Schematic depicting the developmental synaptogenesis and refinement of CF to PC synapses. Four representative phases of the cerebellar circuit maturation are described with the molecular underpinnings involved in the process. Bold molecules indicate molecules implicated and identified in different SCAs in both human and rodent models. GCP, granule cell progenitor; CF, climbing fiber; MF, mossy fiber; GC, granule cell; PF, parallel fiber; BC, basket cell; SC, stellate cell.

The establishment of CF monoinnervation on PCs requires extensive pruning of synapses. An early study in the x-irradiated agranular cerebellum in the rat supports two consecutive phases of elimination: an early GC independent phase (from P7 to around P11 in the mouse) and a late phase (from P12 to around P17 in the mouse) that relies on the correct establishment of PF synapses onto PCs (Crepel et al., 1981). Indeed, impairing PF to PC synapse formation by deleting the glutamate receptor δ2 (GluRδ2) caused a marked reduction in PF synaptic contacts on PC dendrites by the second postnatal week (Kurihara et al., 1997). Additionally, persistent CF-mediated PC polyinnervation (Hashimoto et al., 2001) and its invasion into PF territory i.e., the distal portion of PC dendritic tree was observed (Hashimoto et al., 2001; Ichikawa et al., 2002). Several molecules and signaling pathways participate in CF synapse elimination process. These involve Serotonin 3A receptors (5-HT_3__A_) (Oostland et al., 2013), C1Ql1-Bai3 signaling (Kakegawa et al., 2015), IGF-I-mediated signaling (Kakizawa et al., 2003), mGluR1 (Kano et al., 1997; Levenes et al., 1997), PKCγ (Kano et al., 1995), phospholipase Cβ4 (Hashimoto et al., 2000), semaphorin 7A (Sema7A)-Plexin C1 signaling (Uesaka et al., 2014), and Sema7A-Integrin B1 (Uesaka et al., 2014). Ligand-receptor signaling such as BDNF-TrkB signaling (Bosman et al., 2006; Johnson et al., 2007; Sherrard et al., 2009; Choo et al., 2017), NMDA receptors (Rabacchi et al., 1992; Kakizawa et al., 2000), GluRδ2 receptors (Kurihara et al., 1997), and P/Q type voltage gated calcium channels (Miyazaki et al., 2004; Hashimoto et al., 2011) are also implicated. GABAergic transmission (Nakayama et al., 2012), the immediate early gene Arc (Mikuni et al., 2013), Ca/Calmodulin Kinase IV (Ribar et al., 2000), αCa/Calmodulin Kinase II (Hansel et al., 2006) plays an important role in this process. Lastly, glutamate transporter GLAST (Watase et al., 1998; Miyazaki et al., 2017), the brain-specific receptor-like proteins BSRPs (Miyazaki et al., 2006) and myosin Va (Takagishi et al., 2007) are also involved (see Figure 2 for complete list of molecules involved in this process). Molecular pathways and players determining CF strengthening and synaptic refinement are susceptible to SCA-causing molecules/mutations and several studies have identified problems in CF to PC synaptic transmission (Smeets and Verbeek, 2016). Physiological, morphological and developmental abnormalities of these synaptic inputs can precede PC degeneration (Duvick et al., 2010; Barnes et al., 2011; Ebner et al., 2013; Ruegsegger et al., 2016).

The pharmacological manipulation of the Insulin-like growth factor I (IGF-I) pathway *in vivo* revealed the involvement of this hormone in the early phase of CF synapse elimination and strengthening. Application of exogenous IGF-I to the cerebellum at P8 caused an increase in the percentage of PCs displaying CF-mediated polyinnervation in young adult mice; and no significant effect when the IGF-I signaling was augmented during the late phase of synaptic pruning (P12). Moreover, together with the strengthening of the winner CF, the enhancement of weaker CF-mediated excitatory responses was also observed. In agreement with these results, inhibiting IGF-I signaling by local application of antisera against IGF-I and its receptor decreased EPSCs elicited by activation of the winner CF (Kakizawa et al., 2003). The analysis of transcriptional changes in murine models of SCA1 and SCA7 revealed the conserved downregulation of mRNA coding for the insulin-like growth factor binding protein 5 (IGFBP5) (Beattie et al., 2006), leading to enhanced IGF-I receptor activation probably via increased IGF-I availability (Gatchel et al., 2008). The modulation of the IGF-I signaling pathway by the mutant-ATXN1 therefore could interfere with CF strengthening and synapse elimination. Indeed, CF polyinnervation of young adult PCs and the impaired pruning of somatic CF synapse have been identified in SCA1 (Ebner et al., 2013). Nevertheless, a detailed analysis of the process of CF synapse elimination during cerebellar development is required to underpin SCA1- associated defects in CF wiring and function.

The retrograde BDNF to TrkB signaling in the developing cerebellum contributes to the late phase of CF synapse elimination. The PC-targeted knockout of BDNF resulted in a significant increase in the percentage of PCs showing polyinnervation by CFs at P16-P19, while no difference in innervation was detected prior to this time point. Similar results were obtained when the expression of TrkB was specifically downregulated in CFs (Choo et al., 2017). Accordingly, qRT-PCR analysis of post-mortem cerebellar samples from SCA6 patients revealed a significant decrease in the expression of BDNF transcripts (Takahashi et al., 2012), and an impairment in synaptic elimination of weaker CFs in the Ca_v_2.1[84Q] SCA6 mouse model (Jayabal et al., 2017).

The mGluR1 cascade participates in postnatal maturation of CFs and mice lacking mGlur1 (Kano et al., 1997) or its downstream effectors PLCβ4 (Hashimoto et al., 2000) and PKCγ (Kano et al., 1995) displayed impairments in the late phase of synapse elimination. Importantly, the PC-specific expression of a splice variant of mGluR1 in mGluR1 knockout mice was effective in rescuing the late phase of CF synapse elimination (Ichise et al., 2000). Several lines of evidence indicate that the activation of mGluR1 receptors at PF to PC synapse is a major player at this stage of CF development (Hashimoto and Kano, 2013). mGluR1 signaling defects at PF to PC synapses have been identified in rodent models of SCA1 (Shuvaev et al., 2017), SCA3 (Konno et al., 2014), and SCA5 (Armbrust et al., 2014). The reduced amplitude of the mGluR1-dependent slow EPSCs component (Hartmann et al., 2008), the reduced dendritic calcium transients mediated by the mGluR1-PLCβ-IP3 receptor pathway activation and the impairment of short (Brown et al., 2004) and long-term plasticity (Ichise et al., 2000) have been demonstrated in these models. Furthermore, mGluR1 targeting to the PC dendritic spines was affected and a more diffuse expression of this metabotropic receptor was observed in SCA3 models (Konno et al., 2014) and SCA5 (Armbrust et al., 2014). On the contrary, the increased basal calcium concentration in PCs is likely to be responsible for the enhancement of mGluR1 signaling via a positive feedback loop in SCA2 (Meera et al., 2017).

mGluR1 signaling is a potential target for pharmacological interventions. Acute treatment of SCA1 mice via a single injection of the mGluR1 positive allosteric modulator Ro0711401 resulted in a significant long-lasting improvement of motor performance (Notartomaso et al., 2013). Exploiting mGluR1-GABA_B_ functional coupling to enhance mGluR1 signaling (Tabata and Kano, 2006) was also proven effective in rescuing motor impairment in SCA1 mice (Shuvaev et al., 2017). Moreover, in the doxycycline-regulated conditional transgenic mouse model of SCA1, the improved motor performance of symptomatic mice due to the inhibition of mutant-ATXN1 expression was associated with restored mGluR1 expression levels in dendritic spines (Zu et al., 2004).

Missense mutations in PKCγ, the downstream mGluR1 effector, causes SCA14 (Chen et al., 2003). The expression of the SCA14-associated PKCγ-S119P mutant via *in vivo* lentiviral-mediated transduction of cerebellar neurons impaired CF synapse elimination, resulting in an increased number of PCs exhibiting polyinnervation. Importantly, this deficit in CF maturation appeared only by the expression of the PKCγ mutant during the time window of cerebellar development (Shuvaev et al., 2011). Among the proteins that contribute to the late phase of elimination, the GLAST glutamate transporter, NMDA receptors and αCamKII have been shown to be affected in SCA1. The decreased expression of the GLAST glutamate transporter in Bergmann glia (Cvetanovic, 2015), and the aberrant activation of extra synaptic NMDA receptors (Iizuka et al., 2015) in the cerebellum are involved in disease progression. Moreover, the decreased activation of αCamKII has been observed in SCA1 mouse model (Ruegsegger et al., 2016).

PF to PC synapse formation and transmission are also affected in SCAs. Abnormal PF invasion of CF innervation territory onto PCs is associated with the PC-specific expression of the pathogenic ATXN1-[30Q]-D766 mutant (Ebner et al., 2013); and the reduced strength of PF to PC synapse has also been reported in the SCA1[82Q] mouse model (Hourez et al., 2011). In mixed rat primary cerebellar cultures containing GCs and PCs, transfection of GCs with the SCA27 causing splice variant FGF14b-F150S mutant negatively modulated calcium currents in GCs and impaired PF to PC synaptic transmission, as demonstrated by the reduced EPSCs amplitude evoked in PCs by GCs (Yan et al., 2013). In agreement with these *in vitro* results, a reduced PC responsiveness to PF stimulation was also observed in acute cerebellar slices from the FGF14 knockout SCA27 mouse model, likely caused by a presynaptic deficit in glutamate release (Tempia et al., 2015).

The involvement of altered glutamatergic transmission in SCAs is well established, but the role of GABAergic transmission in these diseases remains poorly investigated. Interestingly, the GABA transporter 1 (GAT1) KO mouse (Chiu et al., 2005) and the vesicular GABA transporter (VGAT) KO mouse (Kayakabe et al., 2013) display poor motor coordination and an ataxic gait. Moreover, the Ax^J^ cerebellar ataxia mouse model is characterized by PCs expressing high levels of the ionotropic GABA_A_ receptor (GABA_A_R) and enlarged IPSCs (Lappe-Siefke et al., 2009). These findings therefore support a possible role of GABAergic transmission in ataxia onset and in disease progression. Indeed, postmortem analysis of brain tissue from SCA1 patients revealed a prominent increase in the BC to PC synapses (Edamakanti et al., 2018).

## Discussion

Key molecules and molecular pathways involved in PC development, activity and cerebellar afferent synaptic organization are affected in SCAs highlighting the possible role of cerebellar development and circuit wiring in the pathobiology of these neurodegenerative diseases. As the earliest manifestation of several SCAs, developmental defects characterize the conserved long silent phase of the pathology. However, their causative vs. protective role in the onset of ataxia and degeneration requires further investigation. Results from the conditional SCA1 mouse model supports their detrimental role, since motor impairment is partially rescued by delaying the expression of mutant ATXN1 until cerebellar development is complete (Serra et al., 2006). Nevertheless, if and how developmental deficits exacerbate the severity of the disease in other SCAs has still to be elucidated and further experiments are required.

A so far poorly explored area in the SCA research field is the investigation of the downstream effects of cortical wiring deficits on the final output stage of the cerebellum that generates the abnormal commands underlying motor impairments. The DCN is the main output of the cerebellum, and it receives GABAergic projections from PCs and excitatory inputs from CFs (Sugihara et al., 1999) and from MF collaterals (Shinoda et al., 1992; Matsushita and Gao, 1997; Matsushita and Xiong, 1997; Wu et al., 1999). Stimulation of the IO mediates the rebound increase in the firing of excitatory DCN neurons following an initial PC-mediated pause in their activity (Hoebeek et al., 2010). DCN activity is also effectively modulated by PC synchronicity that mediates time-locked spiking and rising of the firing frequency as well (Person and Raman, 2012a). Mechanisms of PC synchronizations are not fully elucidated, but experimental evidence supports the role of CF, GC to PC transmission and/or PC collaterals (Person and Raman, 2012b). By affecting the cortical synaptic organization of the cerebellum and PC activity, SCAs molecules could therefore strongly impact DCN activity and cerebellar neuronal coding; the so far identified defects in SCAs predict negative effects on the rebound firing properties of the DCN and PC synchronicity that require further and much needed investigation.

## Author Contributions

FB CP, and SS wrote the review. SS supervised the overall project. All authors read and commented on the final version of the review.

## Conflict of Interest

The authors declare that the research was conducted in the absence of any commercial or financial relationships that could be construed as a potential conflict of interest.
